# More than a defense: salicylic acid's secret role in fruit color

**DOI:** 10.1093/plcell/koag054

**Published:** 2026-03-02

**Authors:** Ved Prakash

**Affiliations:** Assistant Features Editor, The Plant Cell, American Society of Plant Biologists, United States; Department of Plant Pathology, The Ohio State University, Wooster, OH 44691, United States

The vibrant red skin of an apple (*Malus domestica*) is not only a visual delight for consumers but also an important biological trait. The variation in the color of different apple varieties comes from varying proportions of a flavonoid pigment, anthocyanin, in the apple fruit skin. Anthocyanin not only provides coloration but also serves to attract seed dispersers and protect the fruit from the UV radiation and fluctuating temperatures (Steyn et al. 2002; Shang et al. 2011; Zhao et al. 2023). Although the role of ripening hormones such as ethylene and abscisic acid is well documented in promoting pigmentation in fruits, the influence of defense-related hormones in fruit pigmentation has remained less explored.

In a recent study, **Lei Zhao and colleagues (**Zhao et al. 2026**)** examined how salicylic acid (SA) induces anthocyanin biosynthesis (see Fig. 1). Besides the well-established role of SA in plant immunity, the authors found that SA treatment can induce anthocyanin accumulation in apple. This induction was mediated by increased accumulation of *MdMYB1* transcripts, a master transcription factor that increased in 30 min and peaked 1 hour after SA treatment. Hampering the *MdMYB1* expression using tobacco rattle virus–based silencing impaired SA-induced anthocyanin accumulation in the fruit. The authors observed similar results through antisense suppression of *MdMYB1* expression in apple callus, suggesting that MdMYB1 is essential for SA-induced anthocyanin biosynthesis.

**Figure 1:**
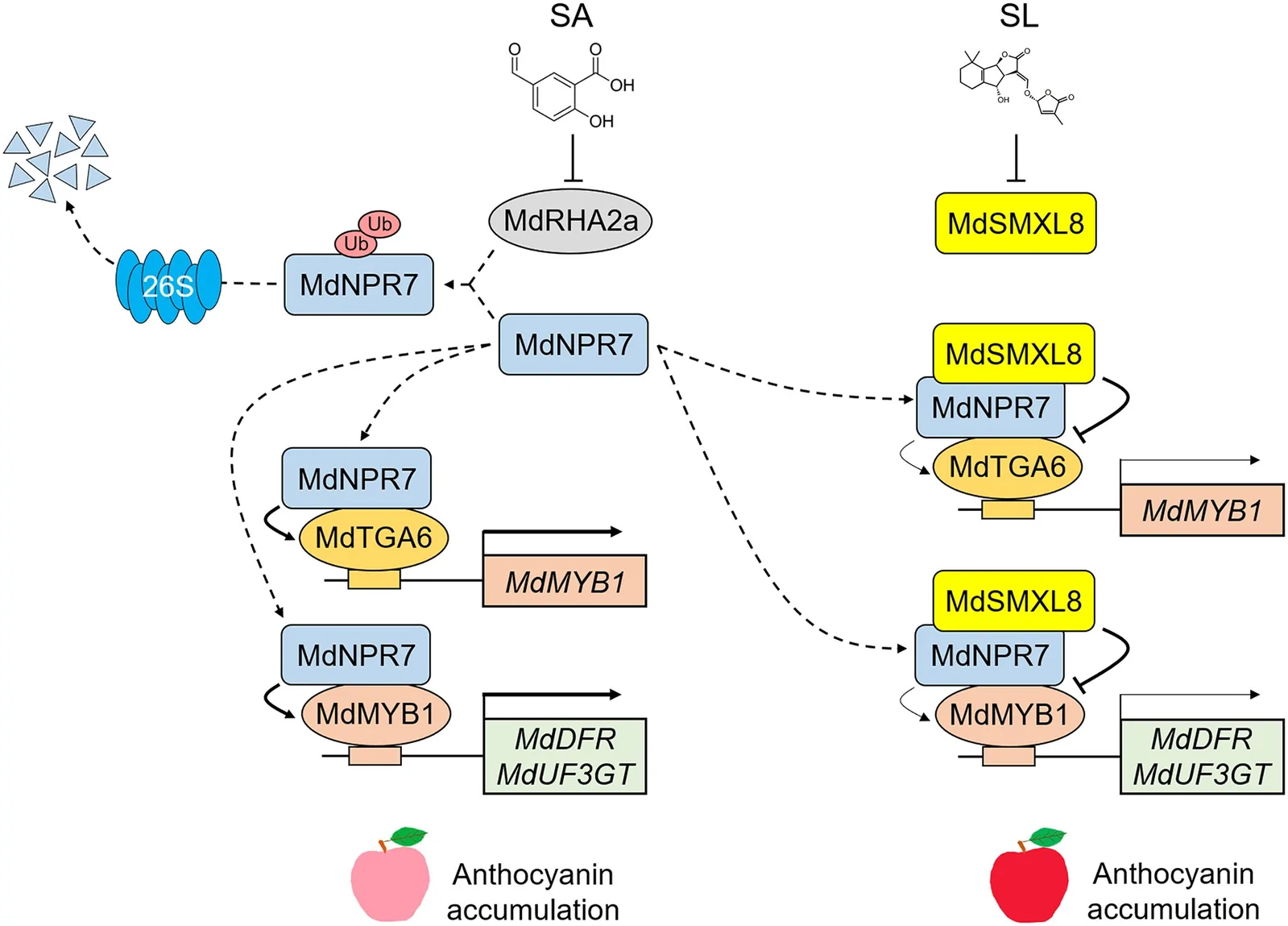
Proposed model illustrating the crosstalk between players of SA and strigolactone (SL) signaling during anthocyanin metabolism leading to fruit color variation in apple. Both SA and SL stimulate anthocyanin biosynthesis through MdNPR7-TGA6-MYB1 module. MdRHA2a and MdSMXL8 act as negative regulators of anthocyanin biosynthesis by affecting MdNPR7 availability for MdTGA6. Adapted from Zhao et al. (2026), Figure 9.

The authors further identified another SA-responsive transcription factor, MdTGA6, which binds to the promoter of the *MdMYB1* gene to activate its transcription. TGA transcription factors belong to the basic region leucine zipper (bZIP) protein family. Using chromatin immunoprecipitation-PCR and electrophoretic mobility shift assays, the authors confirmed the interaction of MdTGA6 with the *MdMYB1* promoter; as well, using GUS reporter and luciferase assays, they confirmed that MdTGA6 activates *MdMYB1*. Importantly, this process requires a copilot, MdNPR7, an apple ortholog of the well-known SA receptor and transcriptional coactivator *NON-EXPRESSOR OF PATHOGENESIS-RELATED GENES 1* (NPR1) (Chen et al. 2021). MdNPR7 physically interacts with MdTGA6, as confirmed by 4 independent methods: yeast 2-hybrid assay, pull-down assay, chromatin immunoprecipitation, and bimolecular fluorescence complementation. The interaction between MdNPR7 and MdTGA6 significantly enhanced the transcription of *MdMYB1*. Additionallly, when the authors silenced *MdNPR7*, they found a visible decrease in anthocyanin biosynthesis in apple fruit as well as in callus, confirming the role of MdNPR7 in promoting anthocyanin synthesis. MdNPR7 was shown to have another crucial function as it interacted directly with the MdMYB1 protein. This physical interaction of MdNPR7 with MdMYB1 enhanced MdMYB1's affinity for the promoters of downstream anthocyanin biosynthesis genes, effectively promoting the anthocyanin-production machinery in apple.

Furthermore, in the absence of SA, the RING-type E3 ubiquitin ligase MdRHA2a targets MdNPR7 for degradation by the 26S proteasome. This constant turnover of MdNPR7 ensures that anthocyanin production remains low under normal conditions. However, when SA is present, it interferes with the MdNPR7 degradation process, allowing the MdNPR7-TGA6-MYB1 module to accumulate and initiate anthocyanin biosynthesis.

Another important finding of the study is integration of SA with strigolactone (SL) hormones in that anthocyanins accumulated more strongly in fruit during co-treatment with SA and SL compared to individual treatments. When SL signaling is activated, a repressor protein, MdSMXL8, is degraded, which allows MdNPR7 to promote anthocyanin production. Thus, MdNPR7 is a central component of the SA-mediated anthocyanin biosynthesis that integrates defensive signals, growth regulators, and metabolic outputs. The findings from this research broaden our understanding of SA from being a defense hormone to also being a versatile regulator of fruit color.

This research identified several targets for molecular apple breeding. By interfering with the stability of MdNPR7 or its interaction with MdSMXL8, breeders may develop apple varieties that have increased coloration and better resilience. This is particularly crucial under climate change because increased temperature can affect pigmentation. Furthermore, the increase in SA-mediated defense may extend fruit life by improved resistance to pathogens. This study not only identifies an intricate molecular mechanism of SA-mediated anthocyanin biosynthesis in apple but also presents a new case for the multi-hormonal control of plant secondary metabolism.

## Recent related articles in *The Plant Cell*:

- An et al. (2024) identified that the SL response factor AGL9 and SL signaling repressor SMXL8 mediate the crosstalk between SL and gibberellin, and this crosstalk regulates anthocyanin biosynthesis in apple.
- Q. Sun et al. (2025) explored ethylene-mediated citrus peel reddening. The authors found that the transcription factor CsERF25 binds to the promoter of the *CsRP1* gene, thus enhancing its transcription. CsRP1 protein then binds to the *CsCCD4b* promoter, activating its transcription and thus leading to reddening of citrus peel.
- Y. Sun et al. (2025) showed that the auxin and endoplasmic reticulum stress pathways determine the inhibition of anthocyanin biosynthesis in grape during high temperature.

## Data Availability

No new data were generated or analysed in support of this research.

## Dive Curated Terms

The following phenotypic, genotypic, and functional terms are of significance to the work described in this paper:

NPR1 Gramene: AT1G64280
NPR1 Araport: AT1G64280

SMXL8 Gramene: AT2G40130
SMXL8 Araport: AT2G40130
