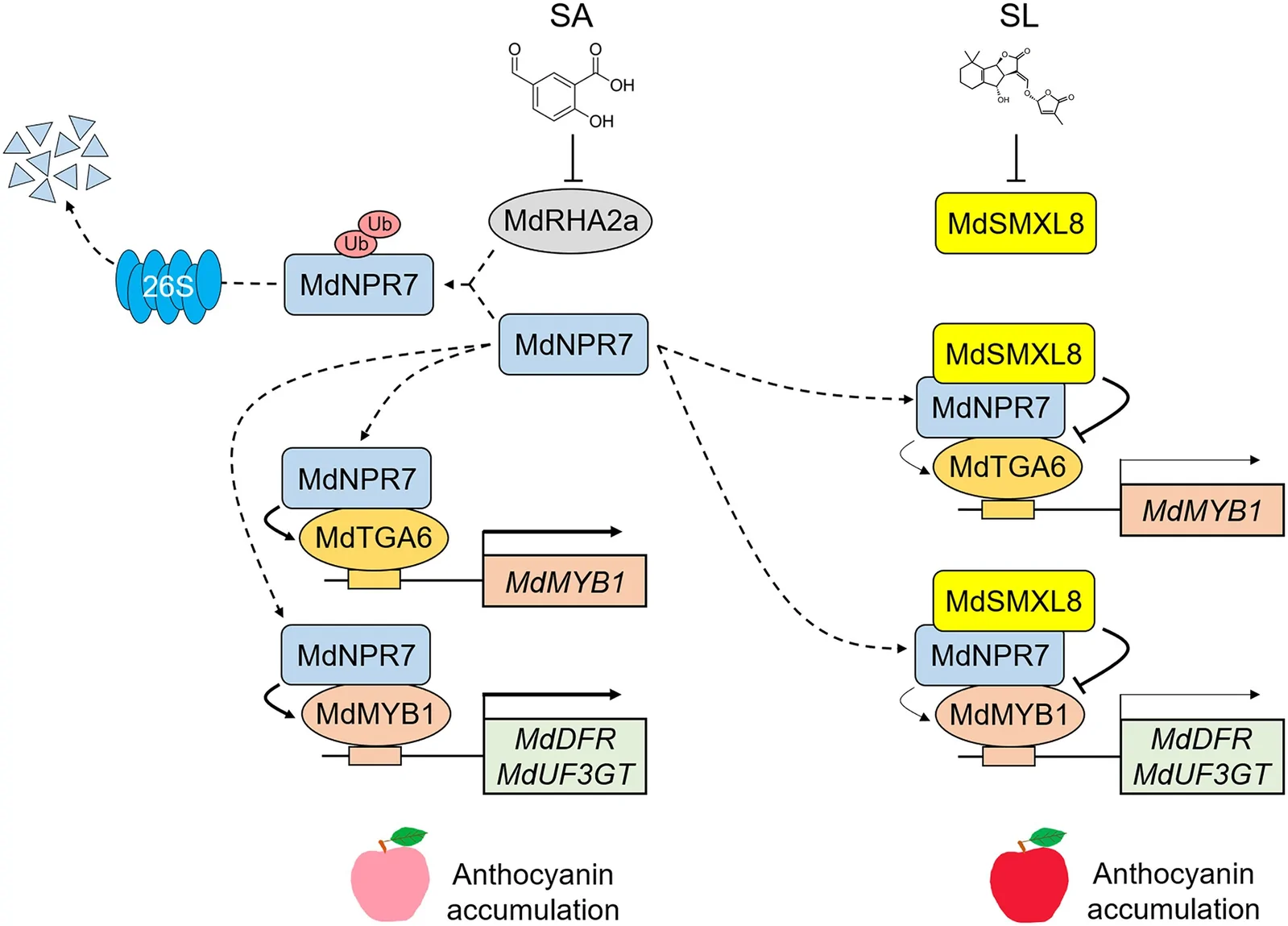# Correction to: More than a defense: salicylic acid's secret role in fruit color

**DOI:** 10.1093/plcell/koag244

**Published:** 2026-09-10

Ved Prakash, More than a defense: salicylic acid's secret role in fruit color, *The Plant Cell*, Volume 38, Issue 3, March 2026, koag054, https://doi.org/10.1093/plcell/koag054

In the originally published version of this manuscript, Figure 1 used the GA structure instead of the SL structure. This error has been corrected and the replacement Figure 1 uses the SL structure.

**Figure:**